# Diagnosis of Ischemic Stroke: As Simple as Possible

**DOI:** 10.3390/diagnostics12061452

**Published:** 2022-06-13

**Authors:** Hana Malikova, Jiri Weichet

**Affiliations:** Department of Radiology, Third Faculty of Medicine, Faculty Hospital Kralovske Vinohrady, Charles University, Srobarova 1150/50, 11000 Prague, Czech Republic; jiri.weichet@fnkv.cz

**Keywords:** MRI, CT, large vessel occlusion, thrombectomy

## Abstract

The absolute majority of strokes in high-income countries, roughly 91%, are of ischemic origin. This review is focused on acute ischemic stroke (AIS) with large vessel occlusion (LVO) in the anterior circulation, which is considered the most devastating subtype of AIS. Moreover, stroke survivors impose substantial direct and indirect costs of care as well as costs due to productivity loss. We review of diagnostic possibilities of individual imaging methods such as computed tomography and magnetic resonance imaging, and discuss their pros and cons in the imaging of AIS. The goals of non-invasive imaging in AIS are as follows: (a) to rule out intracranial hemorrhage and to quickly exclude hemorrhagic stroke and contraindications for intravenous thrombolysis; (b) to identify potential LVO and its localization and to quickly provide guidance for endovascular treatment; (c) to assess/estimate the volume or size of the ischemic core. We suggest fast diagnostic management, which is able to quickly satisfy the above-mentioned diagnostic goals in AIS with LVO.

## 1. Introduction

Stroke is undoubtedly challenging medically and has great social and economic impact. Population-based studies have shown that the incidence of stroke in high-income countries decreased significantly between the years 1970–2000, and that the incidence continues to decrease with a current annual rate of 1–1.5%. Since the year 2000, mortality rates from stroke have fallen in all members of the Organization for Economic Co-operation and Development (OECD) and partner countries, with an average reduction of 52% [1]. The explanation for this is a reduction in risk factors, primarily smoking, and improvement in the quality of medical care [1]. However, some authors have predicted an increase in the absolute number of first-time strokes in 2045, with an estimated increase of 13% due to the aging population [2,3]. Moreover, mortality associated with strokes remains high, as strokes are responsible for 7% of all deaths according to an OECD report published in 2021 [1]. Additionally, stroke survivors impose substantial direct and indirect costs of care as well as costs due to productivity loss [4].

The majority of strokes in high-income countries, approximately 91%, are of ischemic origin [5]. According to the well-known and frequently-used TOAST (Trial of ORG 10172 in Acute Ischemic Stroke Treatment) classification, acute ischemic strokes (AIS) are divided into large vessel occlusion (LVO), small penetrating artery thrombotic strokes (lacunar strokes), cardiogenic embolic strokes, cryptogenic strokes of unknown cause and strokes associated with other causes [6]. Notably, the TOAST classification does not include silent strokes, which have recently garnered greater interest due to progress in cardiac surgery and interventional cardiac procedures. Silent strokes are defined as imaging findings of acute infarction primarily on MRI without neurological manifestation in common bed-side examinations [7,8]. 

This review is focused on AIS with LVO in the anterior circulation, i.e., in occlusion of the internal carotid artery (ICA) and middle cerebral artery (MCA). This severe type of stroke has been of great interest to the neurological community for many years and is considered the most devastating subtype. It has been proven that causal treatment by endovascular reperfusion therapy, primarily endovascular thrombectomy (EVT), is a major determinant of clinical outcome [9]. The DAWN and DEFUSE 3 studies showed that patients, appropriately selected according to ischemic core volume on advanced imaging methods, may benefit from EVT up to 24 h after the onset stroke symptoms [10,11]. However, we must emphasize that the phrase “time is brain” remains valid [12]; time remains a crucial factor in all segments of the diagnostic and treatment workflow. The clinical outcome of stroke patients directly depends on the speed and availability of diagnostic and therapeutic processes. Modern medicine has struggled with stroke for decades; despite all diagnostic and therapeutic progress, research focused on improving stroke diagnosis and treatment remains a work in progress and likely will continue so for the foreseeable future. 

The aim of the present review was to outline the diagnostic possibilities of individual imaging methods in AIS with LVO and discuss their pros and cons. 

## 2. Main Goals of Imaging Methods in the Diagnosis of Stroke

The main aims of imaging methods in AIS are: (a) to rule out intracranial hemorrhage and to exclude hemorrhagic stroke as well as contraindications to intravenous thrombolysis (IVT); (b) to identify potential LVO and its localization; (c) to assess/estimate the ischemic core. 

## 3. First Task: To Rule Out Intracranial Hemorrhage

**Figure 1:**
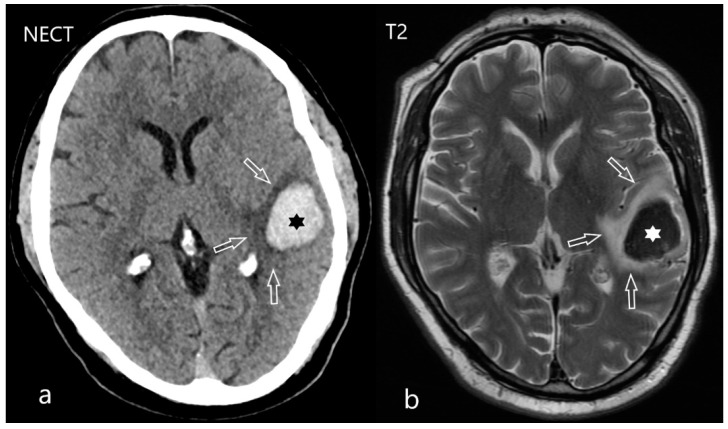
Acute intracerebral hematoma. 57-year-old male was referred with one day aphasia with acute onset. There is an acute intracerebral hematoma (star) on NECT (**a**) and on T2- weighted MRI (**b**) situated in the left temporal lobe. Perifocal vasogenic edema is also present (arrows). The source of hemorrhage was a pial arteriovenous malformation.

**Figure 2:**
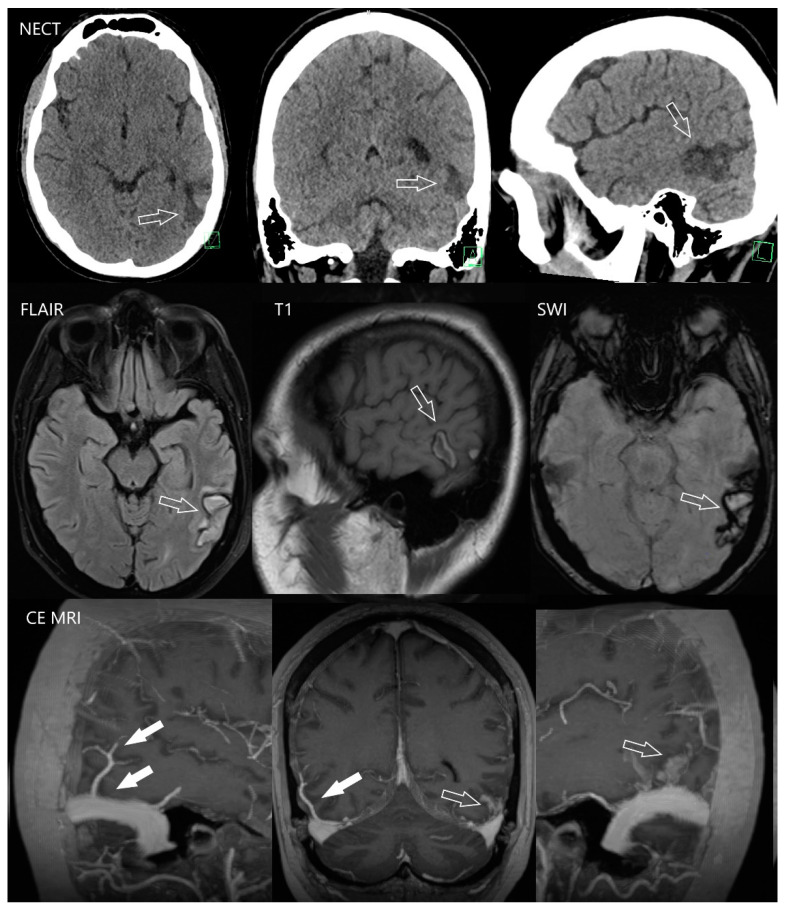
Cerebral venous infarction due to occlusion of the vein of Labbé. 21-year-old male with history of long-term lasting ulcerative colitis treated by vedolizumab and azathioprine. He came to emergency due to the first epilepsy seizure attack in his life. The next relevant data from his history were as follows: Three months before, he underwent embolic occlusion of the femoral artery that was treated by urgent surgery. The source of emboli was not revealed. Two months before, he was treated for acute thrombosis of the femoral vein. NECT (upper row) revealed hypodense region in the posterior aspect of the left temporal lobe (arrows), MRI was indicated to specify the finding. MRI (middle row) depicted the unregular subacute hematoma (arrows) which was suspected from hemorrhagic transformation of venous infarction. On 3D T1 WI (lower row) after gadolinium-based contrast agent application the left vein of Labbé was not present (open arrows). Compare with the right vein of Labbé (full arrows) which has normal appearance.

CT is a basic imaging method in the diagnostic workflow of stroke patients. CT is widely available, could examine nearly all patients included non-cooperating ones, a CT brain scan is very fast, being acquired in seconds. The advantage of non-enhanced CT (NECT) is the lack of absolute contraindications; generally speaking, in the acute setting, pregnancy is not considered a contraindication. For years, NECT has served as the gold standard method for detecting intracranial hemorrhage. NECT, with high sensitivity, is able to identify a small amount of acute blood in the brain parenchyma as well as in the extra-axial spaces such as the subarachnoid, subdural, epidural and intraventricular spaces [13]. However, NECT is not the only method which is sovereign in the diagnosis of acute intracerebral hemorrhage (ICH). MRI, especially with the help of T2 susceptibility-weighted imaging (T2 SWI) and diffusion-weighted imaging (DWI), is able to detect hyperacute/acute ICH with excellent accuracy (Figure 1) [14,15]. However, in some cases, conventional MRI is not able to distinguish between calcifications and hemorrhage; thus, both pathologies are hypointense on T2 SWI and sometimes calcifications may be hyperintense on T1-weighted sequences similarly as subacute hematoma. However, quantitative susceptibility mapping is a novel postprocessing technique that may successfully solve that diagnostic problem [16]. Moreover, in comparison to NECT, MRI has higher sensitivity in the detection of subacute or chronic ICH as well as microhemorrhages (Figure 2) [15]. The sensitivity of NECT in the detection of microbleeds is especially poor [17]. MRI is also more sensitive than NECT in detecting hemorrhagic transformation of infarction (Figure 2) [17].

The detection of aneurysmal subarachnoid hemorrhage (SAH) is not as straightforward as the detection of ICH; there are pitfalls primarily related to time factors as well as the amount of hemorrhage. NECT detection of SAH depends on the attenuation (in Hounsfield units) of blood in the cerebrospinal fluid [18]. This attenuation depends on the amount of blood mixed with cerebrospinal fluid, on the hematocrit and hemoglobin level, and on the level of hemoglobin degradation. The sensitivity of NECT for SAH within first 6 h after stroke event onset is 99% [18]. However, the sensitivity of NECT in the days following stroke onset decreases moderately; after 5 to 7 days from the acute SAH event, the sensitivity of NECT decreases sharply [19]. Therefore, in the case of strong suspicion of SAH, other methods must be applied. One of the methods able to satisfactorily solve this problem is lumbar puncture, which has often been required to show xanthochromia and confirm SAH [19]. However, with the advantage of modern imaging methods such as MRI, invasive lumbar puncture may be avoided. MRI of the brain, with use of T2-weighted fluid-attenuated inversion recovery (T2 FLAIR), DWI and T2 SWI sequences, is very sensitive in the detection of both acute and subacute SAH, with a sensitivity of approximately 98% [20]. The reason for this high sensitivity in the detection of subacute SAH is the ability of T2 FLAIR sequences to show even minimal protein in the cerebrospinal fluid [21]. Moreover, another advantage of MRI is the possibility to identify other potential causes of the patient’s acute status that may mimic stroke, called stroke mimics. There are however some disadvantages of MRI that limit its use as a method of the first choice. MRI scans take considerably longer; currently the duration of a single sequence typically minutes, with complete brain protocols generally requiring about 15 min. Furthermore, there are contraindications to MRI, both relative and absolute, as well as motion and metal artifacts may reduce the yield of scans. 

## 4. Second Task: To Identify Potential Large Vessel Occlusion and Its Localization

**Figure 3:**
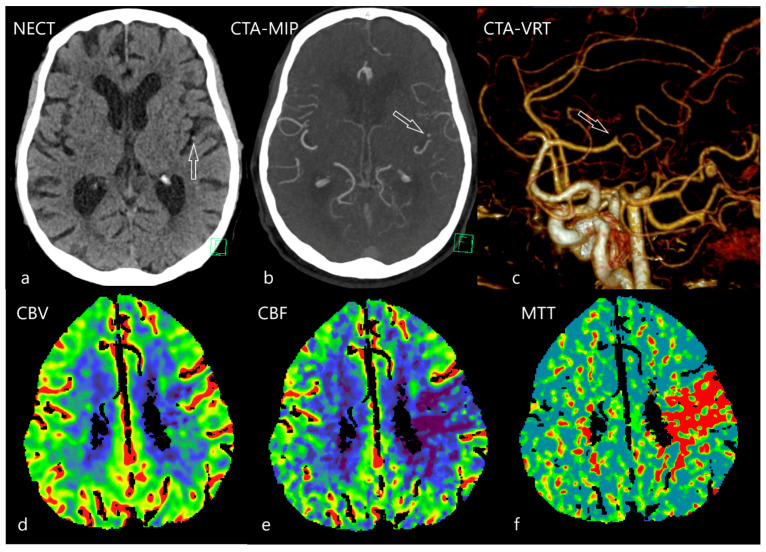
Distal medial cerebral artery occlusion by calcified embolus from mitral valve prosthesis endocarditis. 82-year-old male after mitral and tricuspid valves surgical replacement 11 years ago was admitted to emergency after the fall due to unconsciousness. He suffered from aphasia and the right sided hemiparesis; atrial fibrillation was revealed as well. On NECT (**a**) the small calcified dot was depicted in the Sylvian area (arrow), the same finding (arrow) was apparent on CTA with transversal maximum intensity projection (MIP) 3D reconstruction (**b**), the amputation of one of the arterial branches is seen on 3D volume rendering reconstruction (VRT) [(**c**), arrow]. On CTP (lower row) a region of hypoperfusion is present (**e**), with prolonged mean transit time (MTT) [red area, (**f**)] and decreased cerebral blood flow (CBF) [blue area, (**e**)]; however, the cerebral blood volume (CBV) was not affected [blue area, (**d**)]. Mitral valve prosthesis endocarditis was revealed as the probable source of emboli.

The fact that EVT is currently the standard of care in case of AIS with LVO has been stressed previously. Potential candidates for EVT should be screened for the presence and localization of LVO [22]. CT angiography (CTA) is a fast and suitable tool; a disadvantage of CTA is the necessity of intravenous iodinated contrast agent application, which brings potential adverse effects. Monophasic CTA should be performed from the aortic arch to the brain vertex. There is also the potential for multiphase extension as well as repeating the scan. Monophasic CTA acquired at peak arterial phase typically provides the exact localization of occlusion and a “road map” for EVT navigation. It may also provide basic assessment of collaterals; however, there is the considerable risk of collateral underestimation due to the early time of acquisition. CTA sensitivity for intracranial occlusion detection has been reported as high as 100% [23]. Distal occlusion may, however, be missed by inexperienced raters (Figure 3). According to a recent study by Fasen et al., as many as 20% of LVOs were surprisingly missed on initial CTA evaluation; non-radiologists were more likely to miss LVO compared with radiologists/neuroradiologists, and M2 MCA occlusion was more likely to be missed compared with proximal occlusions [24]. Multiphasic CTA solves these limitations, as equilibrium venous phase and late venous phase may be added to common acquisitions during peak arterial phase (monophasic CTA) [25,26]. Calcified emboli may also be a source of misinterpretation and thus are frequently overlooked [24,27]. The most common potential sources of calcified emboli are calcified aortic stenosis, carotid atherosclerotic plaque and mitral annular calcifications [27]. Careful evaluation of NECT while searching for calcified density in the branches of MCA may be helpful (Figure 3) [27]. Moreover, EVT by stent retriever in the case of calcified emboli is less effective, with greater potential for severe periprocedural adverse events [28]. An important pitfall in the CTA evaluation of extracranial occlusion must be mentioned; hard calcified atherosclerotic plaques may cause difficulties in the differentiation between near-occlusion and occlusion. Near occlusion is often under-reported in clinical practice [29]. 

MRI is another method that may be used in the evaluation of potential LVO and its localization. There are two technical possibilities in MR angiography (MRA); native or unenhanced MRA primarily using the time of flight (TOF) technique, the second is contrast enhanced MRA (CEMRA) with intravenous gadolinium-based contrast agent (GBCA) administration. Briefly, the TOF technique visualizes flow and is based on the phenomenon of the inflow effect of fresh hydrogen protons entering an imaging slice. On the TOF technique the morphology of the vessel is not seen; however, moving blood is depicted. For better visualization, the source data may be reconstructed using maximum intensity projection (MIP) or volume rendering reconstruction (VRT). There are, however, some pitfalls and limitations to TOF. The TOF method is based on the detection of flow; however, slow flow or flow from parallel vessels may be desaturated like stationary (non-moving) tissues and signals from vessels may be lost. Moreover, turbulent flow may cause spin-dephasing and unexpectedly short T2 relaxation resulting again in signal loss. The same may happen in the case of retrograde arterial flow. TOF sensitivity to intracranial stenosis or occlusion detection is lower than CTA sensitivity; 70% sensitivity for stenosis detection, 87% sensitivity for occlusion detection, and approximately 59% positive predictive value for occlusion has been reported [22]. TOF 3D MRA may be as well affected by some T1 hyperintense structures or objects such as hematomas that may be visible and may affect or limit image quality. Moreover, interpretation of the exam may be partly or completely impossible due to patient motion. Another disadvantage is that the sequence generally takes minutes to acquire.

CEMRA is a technique involving T1-weighted spoiled gradient-echo sequences after intravenous administration of GBCA. Single- or multi-phasic data may be acquired. The source data may again be used for MIP and VRT 3D reconstructions. CEMRA removes above-mentioned limitations of different flow artifacts. Disadvantages of CEMRA is the need for GBCA application [30]. A recent study unsurprisingly showed that, in the case of AIS with LVO, CEMRA detects arterial occlusions better than TOF MRA and when combined with other MRI morphological sequences, is better able to assess thrombus length and localization [31]. For a summary, see Table 1.

## 5. The Third Task: To Estimate the Ischemic Core

There are several possibilities for both CT and MRI in the assessment or estimation of potential acute ischemic changes and estimation of the ischemic core. For these purposes, the Alberta stroke program early CT score (ASPECTS) was developed. ASPECTS should be evaluated on NECT. ASPECTS is simply a semiquantitative score that assesses the extent of early ischemic changes in the territory of the MCA [32]. The territory of the MCA is divided into 10 regions, and one point is subtracted for each area where early signs of ischemia are detected, such as hypodensities or blurring. ASPECTS evaluation seems simple; however, there are a number of pitfalls and limitations. Acute ischemic changes within the first hours of stroke onset may be very subtle, as the morphological background of those changes is cytotoxic oedema. The evaluation of these subtle changes on NECT requires an experienced neuroradiologist. It is not surprising that interobserver and intraobserver variability in ASPECTS evaluation is suboptimal [33]. Therefore, automated software based on machine learning was developed to avoid the subjectivity of raters. Initially, some radiologists expressed skepticism with respect to the utility of such software; however, it was soon proven that the automated software is not inferior to evaluation by radiologists [34]. The undeniable advantage of automated software is its speed.

Initially ASPECTS was used for the selection patients that would profit from IVT; thus, ASPECTS grading was proven as an independent predictor of functional outcome [32,35]. Recently, ASPECTS has been used for the selection of subjects for EVT; an ASPECTS score > 6 is a key factor in selecting patients with AIS with LVO in the early time window, up 6 h from stroke onset, that should benefit from acute EVT [9]. It has repeatedly been proven that, in the early time window, ASPECTS ≥ 6 is associated with good clinical outcome and predicts functional independence after EVT [36,37,38]. Conversely, in the case of ASPECTS in the range of 0–5, the benefit EVT is not clear [39]. The role of ASPECTS in selecting AIS patients in the late time window, more than 6 h from last known well (LKW), has not been widely established; the preferred diagnostic imaging method for patient selection is CT perfusion (CTP) or MRI with DWI and MR perfusion (MRP) [10,11]. However, in the literature, we can find some studies that have shown ASPECTS grading as an independent predictor of good clinical outcome in AIS patients in the late or unknown time window, and consider ASPECTS a suitable criterion in selecting patients for EVT in the late time window [40,41].

ASPECTS is not the same as the stroke core volume. In some studies, great variability between specific ASPECTS grade and the cerebral blood volume (CBV) on CTP has been found [36,37]. The explanation is straightforward as each region of the MCA territory in ASPECTS evaluation has a different volume, one point on ASPECTS always means a different brain volume. Finally, some studies have reported that the ischemic core on CTP does not correlate with the definitive infarction volume, at least in the early time window; this topic is discussed in the next paragraphs. However, in a recent study by Nannoni et al. that included 1046 subjects, moderate correlation between ASPECTS and the core volume on CTP in AIS was reported; the correlation was stronger in subjects with LVO and in a subgroup of patients in the late time window [38]. There are several other variables beside the time window and LVO presence that influenced ASPECTS. Pre-stroke statin use positively influences collateral perfusion and is associated with higher ASPECTS scores [38]. In contrast, hyperglycemia at admission is associated with poorer ASPECTS scores [38]. This finding is in concordance with previously-published studies that showed hyperglycemia is associated with early infarct expansion [42]. Unsurprisingly, good collaterals are also associated with higher ASPECTS scores [38].

CTP is an established method used to evaluate the viability of brain tissue. CTP distinguishes ischemic core from penumbra (potentially salvageable tissue) by measuring blood perfusion in cerebral regions following intravenous contrast agent injection. Cerebral blood flow (CBF), CBV and mean transit time (MTT) are basic parameters determined by CTP analysis and are used for ischemic core and penumbra calculations. Software calculates absolute CBF in mL/100 g/min and CBV in mL/100 g for all voxels; however, relative values (rCBF, rCBV) in percentages (%) are also calculated. Standard ischemic core delineation is rCBF < 30% in comparison with the healthy, non-affected hemisphere [43]. Evaluation of CTP data using CT postprocessing software is often a complicated process, therefore automated software based on artificial intelligence was developed to avoid this limitation; its undeniable advantage is its speed.

**Figure 4:**
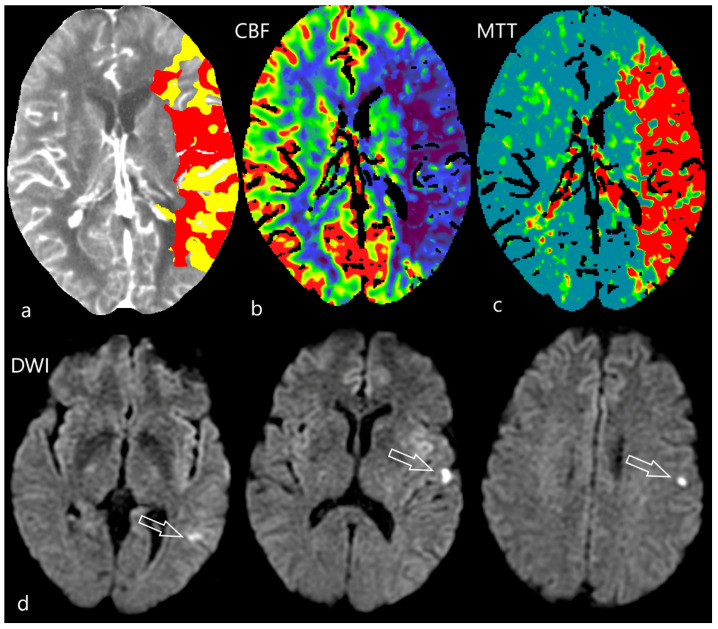
Ghost infarct on CTP. 68-year-old female was referred as acute stroke in the early time window 1.5 h since the last known well. She suffered from right sided hemiparesis and aphasia. CTA confirmed occlusion of the left medial cerebral artery. On CTP [upper row, (**a**)] there is a map with the calculation on penumbra (yellow area) and ischemic core (red area). On the cerebral blood flow map (CBF) a hypoperfusion of the whole region of the left medial cerebral artery was seen [ (**b**), blue area], mean transit time (MTT) was prolonged [(**c**), red area]. Endovascular treatment was promptly indicated. The second day after endovascular mechanical thrombectomy, she was without neurological deficit and was sent to MRI follow-up [lower row, (**d**)]. On diffusion weighted images (DWI) only 3 very small acute ischemic lesions were found (arrows).

Although CTP has been used for decades, its results were often controversial [44]. However, the DAWN and DEFUSE 3 trials figuratively resuscitated CTP, as they used CTP-calculated penumbra volume as a predictive factor for clinical outcome in patients treated with EVT in the late time window [10,11]. However, as was mentioned above, there is evidence that the ischemic core on CTP poorly correlates with the definitive ischemic volume in patients with AIS with LVO after successful urgent EVT, at least in the time window up to 6 h from AIS onset [43,45,46]. Moreover, some authors have reported significant overestimation of initial core volume on CTP, which was romantically called a “ghost infarct” (Figure 4) [47,48]. Despite the above mentioned, DAWN or DEFUSE 3 indication criteria for EVT in the late time window are still used. However, there are studies that bring some data implying that we can possibly stand without CTP. Desai et al. analyzed clinical outcomes of subjects who did not meet DAWN and DEFUSE 3 inclusion criteria and received off-label EVT in the late time window; 30% of treated patients achieved functional independence at 3 months [49]. Moreover, Alexandre et al. treated 49 patients with LVO in anterior circulation in the time window longer than 6 h and did not use CPT at all [50]. In 77% of subjects, recanalization was successful and 18 of 49 treated patients achieved functional independence at 3 months after treatment [50]. The possibilities with MRI are similar to CTP, and MRP is also one of the methods available in the diagnostic spectrum. MRP is based on the detection of signal decrease due to the susceptibility effect of GBCA during the passage GBCA through the intracerebral vessels. The signal decrease observed per voxel is directly proportional to the concentration of gadolinium in the vessels; rCBV, rCBF, MTT and TTP may be calculated from the source data and used for perfusion map construction [51]. 

MRI with DWI and T2 FLAIR sequences is definitively the best method in acute ischemic core volume assessment; their sensitivity and specificity have been repeatedly proven. DWI detects hyperacute ischemic changes at the stage of cytotoxic edema, which appears at the moment of Na^+^/K^+^ ATPase failures, when water molecules translocate from the extracellular to the intracellular space. Moreover, in cytotoxic edema the volume of the cell increases and extracellular space decreases, restricting the free diffusion of water molecules. Hyperacute ischemic changes are detectable as early as minutes to hours from arterial occlusion [51]. The area of high signal intensity on DWI does not unequivocally mean ischemic core, as cytotoxic edema itself may be reversible [7]. Later when cell membrane disruption develops and vasogenic edema appears, not only DWI but also T2 signal increases and positivity on T2 FLAIR appears, which indicates the onset of irreversible ischemic changes [51]. ASPECTS could be also evaluated by the same way as on NECT on DWI that precisely depicts the extend of acute ischemia, this evaluation is simply called DWI-ASPECTS and has also been incorporated into clinical practice [52]. For a summary, see Table 2.

## 6. Factors Affecting the Ischemic Core

In AIS with LVO, the ischemic core and the time from stroke onset or time from LKW to treatment are still considered the 2 most important variables that influence clinical outcomes after EVT. Ischemic core growth is a key factor that affects the benefit of EVT, including delayed EVT. Recently, ischemic growth rate (IGR) was introduced in the literature [53]. In their multicentric study, Sarraj et al. calculated the early ischemic growth rate (EIGR) in AIS patients with LVO in the anterior circulation. EIGR was calculated from the following parameters: ischemic core volume calculated from CTP/time from LKW to imaging. Subjects under 10 mL/h EIGR were considered slow progressors, and above 10 mL/h fast progressors. EIGR independently correlated with functional independence after EVT. Slow progressors achieved better clinical outcome and had 3.5 times greater probability of achieving functional independence (mRS 0–2). Each 5 mL/h EIGR increase led to a 14% decrease in the probability of good clinical outcome. Fast progressors had higher mortality. The authors observed similar results in both early and late time windows. Better collaterals were strongly associated with slow progression of the ischemic core [53]. Previously-mentioned studies that reported poor correlation between the ischemic core on CTP and definitive infarct volume after EVT may suggest that CTP, mainly in the early time window, is not helpful. However, the multicentric study by Sarraj et al. reported different results and they evaluated patients with both time windows as well as patients that were treated both with EVT and medically [53].

**Figure 5:**
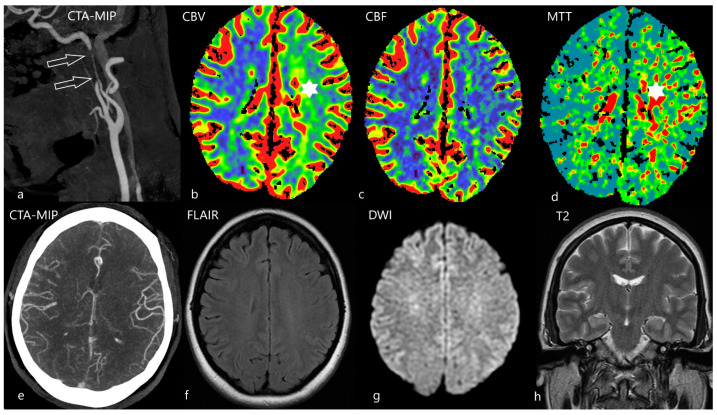
The effect of symmetric collaterals. 40-year-old female referred to emergency for unclear weakness of the right sided limbs and very discrete expressive aphasia that was mentioned by the patient herself since the previous evening. On the CT angiography (CTA) with maximum intensity projection (MIP), stenosis of the left internal carotid artery was found (arrows); dissection was suspected (**a**). CTP revealed apparent discrete signs of hyperperfusion on cerebral blood volume [CBV, (**b**), star], no abnormalities were apparent on cerebral blood flow [CBF, (**c**)], mean transit time (MTT) was slightly prolonged [(**d**), star]. Collaterals on CTA with MIP reconstruction were symmetric (**e**), on MRI scan (**f**–**h**) there were no signs of acute ischemia. She was treated by wall-stent insertion and the next day she was released on home care without any neurology symptoms. The source of dissection was not revealed.

**Figure 6:**
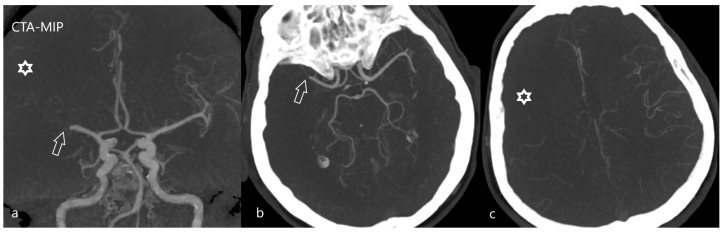
Malignant type of collaterals. CT angiography with maximum intensity projection reconstruction (CTA-MIP) depicts malignant collaterals in a patient with the occlusion of the right MCA [(**a**,**b**)—arrow]. Leptomeningeal collateral vessels are insufficient, peripheral arterial branches are not visible [(**a**,**c**)—star; compare with normal filling of vessels on the healthy left side].

Additional evidence for the importance of collaterals in IGR was published by Regenhardt et al. [54]. Collaterals as alternative vessels in AIS with LVO consisting primarily of circle of Willis and pial-pial leptomeningeal anastomoses are crucial in compensating reduced blood flow in the brain parenchyma. Several suggestions for collateral evaluation, using mono-phasic or multiphasic CTA or CTP, have been published [55,56]. Regenhardt et al. simply assessed collaterals as symmetric (bilateral symmetric vessels nearly without detectable reduction in opacification on the affected side), malignant (presence of very poor pial collaterals) and others; see Figure 5 and Figure 6 [54]. They retrospectively evaluated patients from a previous trial from 2007–2009; patients with AIS with LVO were not treated by EVT and underwent diagnostic CTP and then at least 3 MRI scans during the next 48 h. The authors evaluated IGR and compared it with the pattern of collaterals. They concluded that a symmetric collateral pattern was common and highly specific for slow IGR and smaller initial core volume. They concluded that good collaterals probably strongly influenced the EVT benefit in the late time window [54].

## 7. Conclusions

The diagnostic possibilities of both imaging methods that may be used in AIS were reviewed. Both methods have certain limitations and pitfalls; however, in comparing the sensitivity and specificity of both non-invasive methods, MRI should be preferred. MRI is able to diagnose any type and any age of hemorrhage, appears to be the best method in ischemic core evaluation, and CEMRA has the same sensitivity for arterial occlusion detection as CTA. Moreover, the big advantage of MRI is the possibility of diagnosing stroke mimics. However, it is often stressed that the accessibility of MRI in acute scenarios is limited in some institutions. Moreover, there are some absolute contraindications that are life-threatening for patients and must not be overlooked. The patient’s cooperation is needed, or motion artifacts could completely destroy any MRI examination. If we accept the hypothetical that MRI is accessible and we are able to cope with possible contraindications, one most important question remains: Do we have enough time for MRI? A complete MRI scan included CEMRA may require about 15 min. Time of scan acquisition depends on the scanner field strength and on the technical parameters of sequences. However, we must also consider additional time for patient and equipment preparation, time for excluding contraindications, etc. A further consideration is: How much does one wasted minute cost? In the worst-case scenario, it is estimated that in AIS with LVO every minute costs 1.9 million neurons and 12 km of myelinated fibers; one wasted hour costs 120 million neurons and 714 km of myelinated fibers lost and 3.6 years of accelerated aging [12]. Fortunately, it was proven that not each scenario in AIS is so severe and the individual patient’s clock ticks differently [53,54]. It does not change the undisputable fact that NECT and CTA are very fast and straightforward, and with the help of artificial intelligence, basic evaluation suitable for treatment decision making is relatively fast. In our opinion, NECT for hemorrhage detection and ASPECTS evaluation, and CTA for occlusion detection, is all that is needed in the acute setting especially in the early time window … Although recent guidelines and recommendations favor CTP or MRI for the ischemic core evaluation in the late and unknown time window [10,11]; there are some data that suggest that we probably could manage without it and stick only to ASPECTS and CTA [39,40,49,50]. From our personal experience with ASPECTS > 6, additional significant findings on CTP or MRI are rather unlikely. Although the individual patient’s clock ticks differently as was mentioned above, still each minute must be counted in the management of stroke, because we never precisely know how fast the ticking is and will be in the time that follows. Moreover, CTP means additional iodinated contrast agent and radiation burden and is probably of little use in the EVT decision-making process up to 6 h since LKW; with mounting evidence of overestimation of the ischemic core volume on CTP, relying on CTP results could lead to a substantial risk of excluding some patients from EVT. This does not mean, however, that CTP should be completely avoided in the diagnostic management in AIS. It may be diagnostically helpful in some unclear cases and is still recommended in the late time window. It is necessary to stress that CTP and MRI play an important role in research and help us to understand the pathophysiological mechanism of stroke and its treatment. However, in our opinion, advanced techniques in daily and routine practice are unnecessary. 

## Figures and Tables

**Table 1:** CTA and MRA in the detection of extracranial and intracranial occlusion.

		**CTA**		**MRA**	
		**Monophase**	**Multiphase**	**TOF 3D**	**CEMRA**
**Extracranial occlusion**	Sensitivity	Generally high		Limited	High
	Pitfalls	Misinterpretation of hard calcified stenosis/occlusion		Overestimation/ underestimation	
**Intracranial occlusion**	Sensitivity	High	Generally high	Satisfying	High
	Pitfalls	Risk of underestimation in M2 MCA	-	Risk of overestimation Metal and motion artifacts	Metal and motion artifacts
**Collateral assessment**		Possible	Recommended method	Limited	Possible
**Major disadvantages**		Iodine contrast agent burden Radiation burden	Iodine contrast agent burden Radiation burden >single phase	More time needed >CTA or CEMRA Patient cooperation needed	GBCA burden Patient cooperation needed
**Source of pitfalls**		Calcified distal emboli Hard calcified plaques Inexperienced raters		Flow artifacts T1 hyperintense objects Metal and motion artifacts Inexperienced raters	Inexperienced raters Metal and motion artifacts

CEMRA, contrast enhanced magnetic resonance angiography; CTA, computed tomography angiography; M2 MCA, M2 part of the medial cerebral artery; MRA, magnetic resonance angiography; TOF, time of flight.

**Table 2:** CT and MRI in the ischemic core evaluation in the early time window.

	ASPECTS/ NECT	CTP	MRI: DWI Alone/DWI ASPECTS	MRI: DWI + FLAIR
**Core quantification**	Semiquantitative	Quantitative	Semiquantitative	Semiquantitative
**Correlation of core volume with definitive infarction after EVT**	Limited	Limited	Risk overestimating ischemic core	High sensitivity for definitive core assessment
**Use as predictor of clinical outcome after EVT**	Proven	-	-	-
**Pitfalls/risks**	Underestimation of discrete findings; Overestimation in case of chronic ischemic changes; AI programs helpful.	Risk of core overestimation (“ghost infarct”)	Risk of core overestimation due to reversible cytotoxic edema	-
**Overall limitations**	Radiation burden	Additional radiation burden; Additional iodine contrast agent burden.	More time than CT is needed; Exclusion of contraindications needed; Availability limited; Patient’s cooperation is needed.	

ASPECTS, Alberta Stroke Program Early CT Score; AI, artificial intelligence; CT, computed tomography; CTP, computed tomography perfusion; DWI, diffusion weighed imaging; EVT, endovascular treatment; FLAIR, fluid-attenuated inversion recovery; NECT, non-enhanced computed tomography; MRI, magnetic resonance.
